# Supplementary material for: Preparation of Enzyme-Soluble Swim Bladder Collagen from Sea Eel (Muraenesox cinereus) and Evaluation Its Wound Healing Capacity
Source: Mar Drugs. 2023 Oct 3;21(10):525. doi: 10.3390/md21100525 (PMC10608547; doi:10.3390/md21100525)
Supplement: Supplementary file 1 [file marinedrugs-21-00525-s001.zip › marinedrugs-2585917-supplementary.pdf]

Supplementary data

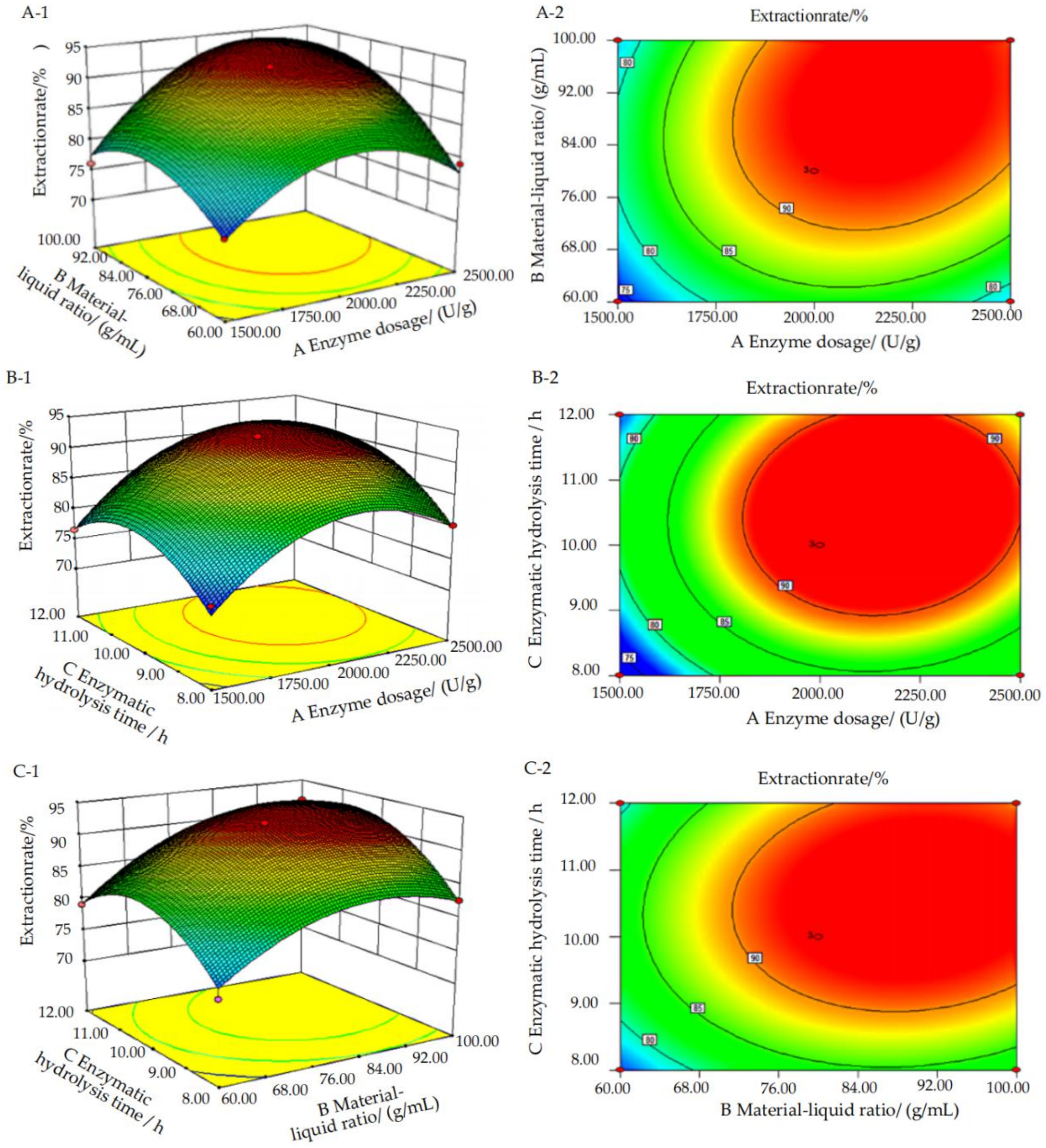

Figure S1. Response surface graph (A-1, B-1 and C-1) and contour graph (A-2, B-2 and C-2) show the interaction between the extraction rate of collagen and the amount of enzyme (A), the ratio of material to liquid (B) and the hydrolysis time (C)

Table S1. Independent factors and their levels used in the response surface design.

Independent Factors	Symbol	Level of Factor		
		-1	0	1
Enzyme concentration / (U/g)	A	1500	2000	2500
Solid-liquid ratio / (g/mL)	B	1:60	1:80	1:100
Hydrolysis time / h	C	8	10	12
